# What are the training and support needs for homelessness hostel staff supporting older residents with memory and cognitive problems? A qualitative study

**DOI:** 10.1186/s12913-025-12246-2

**Published:** 2025-01-21

**Authors:** Luna Zalc, Sophie N. Gaber, Penny Rapaport

**Affiliations:** 1 Division of Psychiatry, University College London, London, England; 2 Department of Women’s and Children’s Health, Healthcare Services and E-Health, Uppsala University, Uppsala, Sweden; 3 Department of Healthcare Sciences, Marie Cederschiöld University, Stockholm, Sweden; 4 London, England; 5 North London Mental Health Foundation NHS Trust, London, England

**Keywords:** Hostel staff, Memory problems, Cognitive problems, Ageing, Homelessness, Inclusion health, Qualitative

## Abstract

**Background:**

An increasing number of older people are experiencing homelessness and memory problems, many of whom are supported in temporary hostel accommodation. This can be a challenge for hostel staff who may not have adequate training and support but who often support those with significant memory impairment in their day-to-day work. The study aimed to investigate the training and support that hostel staff require to meet the needs of older hostel residents experiencing memory and cognitive problems, and thus enhance hostel resident quality of life and well-being, considering what additional knowledge, skills, and support hostel staff need to achieve this.

**Methods:**

In this qualitative study, we conducted inductive reflexive thematic analysis of semi-structured interviews with health and social care practitioners (*n* = 17) and hostel staff and managers (*n* = 15). Participants were recruited from six homelessness hostels, one specialist care home, and National Health and Local Authority Services in England.

**Results:**

We identified three main themes: (a) *Emotional and psychoeducational support needs within hostel staff teams*, including training on memory and cognitive problems, responsive behaviours, communication strategies, and reflective spaces for hostel staff to manage their emotional responses (b) *Training and support needs to deliver a person-centred approach*, including tools and support packages to enhance hostel residents’ independence, multidisciplinary meetings to evaluate hostel residents’ needs, and communication strategies tailored to hostel residents’ understanding and (c) *Support to facilitate collaboration with external agencies,* including training on professional language, capacity assessment, dementia case studies, improved communication skills to bring external services into hostels, and reflective practices to advocate for hostel residents with dual diagnoses.

**Conclusions:**

The present study highlights how hostel staff could benefit from targeted training in emotional support, person-centred approaches for older hostel residents with memory and cognitive problems, and collaboration with external services. Overall, the current study contributes to understanding of the need for tailored training and support methods within hostels to provide higher quality support to a population that is frequently overlooked.

**Supplementary Information:**

The online version contains supplementary material available at 10.1186/s12913-025-12246-2.

## Background

Worldwide, an estimated 100 million people experience homelessness, and older individuals within this group are particularly prone to memory problems compared to those who are not homeless [1]. While this study draws on international research from the US and Europe to provide context, it was conducted entirely in England setting, and predominantly in London, examining the support needs of hostel staff working with older hostel residents experiencing memory and cognitive issues. Studies in the UK indicate that between 5.1% and 7.7% of the population has experienced homelessness at some point in their lives [2]. Moreover, 50% of the US homeless population is 50 and over [3], and the homeless population over 65 is predicted to triple by 2030 [4]. Although specific England statistics are limited, similar trends have been observed, with reports highlighting an increase in older individuals experiencing homelessness. Factors such as loss of rented accommodation and changes in the housing market have contributed to an increase in rough sleeping in England, with numbers nearly tripling since 2010 [5]. Additionally, homelessness and prolonged periods of rough sleeping can accelerate aging and exacerbate health conditions typically associated with older age, making individuals aged 55 and above particularly vulnerable [6]. These trends underscore the growing need for supportive measures tailored to older adults, not only in the US but globally, including in England, where our study is focused. Alzheimer’s disease and related dementias (ADRD) and cognitive impairment are more common in those who have experienced homelessness than those who have not [7]. Among people who have experienced homelessness, ADRD is particularly prevalent between the age of 55 to 65 [8]. Efforts to improve the lives of people experiencing homelessness must consider and address memory problems, as these issues can hinder access to healthcare services, further impacting well-being and overall health [2]. This is especially true for older adults, where memory problems are often overlooked or misattributed to substance abuse, creating a pervasive barrier to receiving appropriate healthcare [9]. This issue is worsened by the lack of trust and feeling of being unheard in the healthcare system, intensified by the transience and instability of homelessness [9]. In turn, these difficulties are challenging for hostel staff, who are integral to supporting hostel residents’ health needs.

Homelessness, as defined by the 2005 European Typology of Homelessness and Housing Exclusion (ETHOS), includes rooflessness (sleeping rough), houselessness (temporary institutional or shelter stays), or inadequate housing [10]. Although this study references some international perspectives, it primarily focuses on hostels in England, which are typically run by the charity sector and by non-clinically trained staff, who provide temporary accommodation to individuals experiencing homelessness, particularly those with complex needs. Hostels are generally not commissioned to provide care. They aim to provide a safe living environment while supporting the search for permanent and stable housing solutions [11]. With the lack of stable housing, especially for older adults experiencing memory problems, hostel staff play a crucial intermediary role in supporting hostel residents’ health needs. However, limitations in understanding how best to support older people experiencing memory and cognitive problems and in identifying what can help hostel staff to achieve this, such as training and support for hostel staff themselves, can inhibit hostel staff effectively address these challenges [12].

People experiencing homelessness have multiple complex health and social care needs, including physical and mental health issues, substance abuse, and cognitive impairments [2, 13–15]. Nevertheless, memory services generally exclude those with alcohol﻿-related cognitive impairments [15, 16]. Additionally, inadequate external support further challenges the ability of hostel staff to assess hostel residents’ capacity and to advocate for the hostel residents’ needs within the healthcare system [15, 16]. Furthermore, hostel residents with memory and cognitive problems face challenges transitioning out of hostels [17, 18]. This problem is compounded by a 26.3% decline in available beds since 2010 in England, with over 70% of accommodation services, including hostels, unable to accept new hostel residents [19]. This decrease is partly attributable to increasing care demands and inadequate hostel staff training to provide necessary support [19, 20]. With a declining number of available beds [19], hostel staff experience pressure to prioritise support for hostel residents without memory and cognitive problems, as those with memory and cognitive difficulties often require more intensive support and face greater challenges in transitioning into suitable accommodation [17, 21].

### Approaches to staff training and support in homelessness hostels

Hostel staff in the United Kingdom (UK) receive minimal training [22]. Psychologically Informed Environments [23] have been introduced to improve hostel staff training and increase the psychological well-being of hostel residents experiencing homelessness. Psychologically Informed Environments focus on adapting hostel settings to meet hostel residents’ emotional needs by encouraging reflective practices among staff, helping them respond thoughtfully to challenges and create a safe, supportive environment. This approach promotes continuous learning, improving the quality of support and reducing burnout [23]. Although trauma is highly prevalent among individuals experiencing homelessness, it is not yet clear if Psychologically Informed Environments target the unique needs of these individuals who additionally experience memory and cognitive problems [22]. Interprofessional Education can facilitate collaboration and acknowledge emotions and attitudes among diverse health and social care practitioners [24], which may benefit hostel staff by promoting their understanding of memory and cognitive problems among older people and addressing stigma and misinformation [25]. Health and social care practitioners, such as social workers, nurses, and general practitioners, typically work in external services, including hospital teams supporting individuals experiencing homelessness or memory clinics for older adults. They provide specialised support for hostel residents based on their formal clinical training, but are not involved in their day-to-day care [16]. Hostel staff, by contrast, manage residents’ daily needs and safety within the hostel environment. Together, these roles form a collaborative approach, with health and social care practitioners focusing on clinical and specialised interventions and hostel staff ensuring practical and day-to-day support [16, 17]. For individuals with memory and cognitive problems, Person-Centred Care or more broadly person-centred approaches, and the Positive Approach to Care, are frequently used alongside Interprofessional Education and Psychologically Informed Environments to provide comprehensive care [26]. The Positive Approach to Care framework for health and social care practitioners and caregivers provides training to address the needs of individuals with dementia, by building communication and trust [26, 27], helping to provide individuals with dementia with an enhanced sense of control over their daily lives [27]. Furthermore, education on memory and cognitive problems might improve care providers’ attitudes and practical skills [28]. Person-Centred Care can improve care providers’ attitudes, behaviour, and communication [29, 30], emphasising the uniqueness of individuals with memory problems and promoting their well-being. Understanding each hostel resident’s needs requires good communication strategies and care management services [14].

### Challenges to integrating psychosocial approaches

Utilising a recovery-focused model can help people experiencing homelessness achieve greater independence and secure permanent housing [9]. Staff often lack the specialised training and resources readily available in hospitals or clinics [31] and face multiple systemic barriers [22]. However, utilising a recovery-focused model has been criticised for excluding people experiencing memory and cognitive problems due to its emphasis on self-care and independence, creating barriers for this population with complex needs [32]. Therefore, understanding staff training and support needs is necessary to meet hostel resident needs. Providing hostel staff with the skills and resources they need to support older hostel residents with memory problems could help them to make changes to their day-to-day interactions with hostel residents, which may support the development of more inclusive care models tailored to the needs of individuals with complex challenges.

### Addressing the knowledge gap

Manthorpe et al. [17] highlighted challenges for hostel residents experiencing memory and cognitive problems, including safety concerns, staff turnover, the assumption that external services have prior knowledge of homelessness, and training for hostel residents with responsive behaviours. Despite existing psychological approaches in healthcare, there is a notable gap in understanding the specific knowledge and skills required by UK hostel staff to address the needs of older people experiencing memory and cognitive problems in the context of homelessness, exacerbated by limited training and support resources [22]. This knowledge gap impedes appropriate care and hinders the development of a specialised staff workforce equipped to support this vulnerable population. To address this knowledge gap, the study aimed to investigate the training and support that hostel staff require to meet the needs of older hostel hostel residents experiencing memory and cognitive problems, and thus enhance hostel resident quality of life and well-being, considering what additional knowledge, skills, and support hostel staff need to achieve this.

## Methods

### Study design

This qualitative study was part of the broader HOME project, which explored the care and support needs of older people experiencing memory problems and homelessness [9]. As a secondary analysis, this research focuses specifically on the perspectives of hostel staff regarding their training and support needs. The primary data, which included interviews with hostel residents, was collected and analysed by the co-authors and is reported in an earlier publication [9]. Qualitative research can delve into individuals’ experiences, perspectives, and meanings in a given environment [33]. This approach was useful for examining complex issues associated with memory and cognitive problems faced by the hostel staff in their role of supporting hostel residents with those challenges.

### Study setting, participant sampling, and recruitment

Participants included 17 health and social care practitioners who commission or provide support for older people experiencing homelessness with memory problems and 15 hostel staff and managers who regularly work with them. Hostel staff were selected from six complex needs hostels and one specialist care home in London run by five voluntary service providers. As all hostel-based data collection was face-to-face, we were limited to hostel locations which could be accessed within two hours of the research team’s base. Health and social care practitioners were accessed via existing networks, from participating voluntary organisations and NHS and local authority (LA) services in London, Sussex and Yorkshire, this was possible as these interviews were conducted remotely. Purposive sampling was used [33] ensuring demographic diversity, including age, professional role, gender, and previous work experience. Understanding the perspectives of health and social care practitioners, key collaborators in providing support services, can shed light on potential barriers and disparities in the current healthcare system for people experiencing homelessness with memory and cognitive problems.

Thirty-two semi-structured interviews were conducted. After recruiting hostel managers, the research team held face-to-face team meetings to explain the study’s purpose, distributed information sheets and invited hostel staff to a face-to-face interview, while health and social care practitioners received an information sheet and invitations to online meetings and interviews via email. The health and social care practitioners were interviewed online, while the hostel staff were interviewed in London-based complex hostel settings. These hostels support residents with co-occurring mental health issues, cognitive impairments, substance misuse, and other health or social challenges, although they do not provide direct health or personal care input. Meetings with hostel staff and health and social care practitioners were conducted to discuss the study, answer questions, and take audio-recorded informed consent. All participants received vouchers as a sign of gratitude.

### Data collection

Data were collected through semi-structured interviews using a semi-structured interview schedule (see Additional file 1). Since semi-structured interviews are flexible, participants can share more specific information and build trust without being constrained by a pre-existing questionnaire. It can also capture details about participants’ perspectives, roles, and obstacles [34]. Health and social care practitioners interviews were conducted via video calls, while hostel staff interviews were conducted in person in private offices in the hostels. Interviews were audio-recorded and anonymised by the project team. Full details on recruitment and data collection are available in our earlier published study [9].

### Data analysis

Data were analysed using reflexive thematic analysis [35]. We imported and organised recordings in NVivo12, where they were transcribed verbatim, checked for accuracy and then coded for analysis. We used an inductive approach to analyse our results, allowing themes and patterns to emerge directly from the data without any pre-set analytical concepts [36].

LZ iteratively reviewed the interviews to familiarise herself with the data and performed initial coding to capture ideas and insights as they naturally surfaced. After multiple readings, we created a coding framework based on the themes and patterns that emerged inductively, systematically structuring it into relevant categories [36]. We investigated the data similarities and differences, putting together related information within nodes as patterns naturally emerged, constantly comparing and refining our codes, themes, and subthemes as new insights developed inductively from the data [35, 36]. The coauthors collaborated through ongoing and extensive discussions to ensure a comprehensive coding scheme [37]. The coding process involved carefully reviewing all 32 interviews multiple times, refining codes until no new themes or insights were emerging. Coding ceased at this point, as the repeated review across all interviews ensured that the main topics were fully explored and that each theme captured a deep, nuanced understanding of participants’ experiences, providing data richness [36, 38].

The themes were developed inductively from the data and later interpreted in relation to the study’s aims. We supported our interpretation of the findings with quotes from hostel staff and health and social care practitioners. Furthermore, LZ sought peer debriefing from the coauthors (qualitative research experts) to review the many interpretations, overcome biases, ensure validity, and demonstrate trustworthiness [39].

By analysing perspectives of health and social care practitioners as well as hostel staff and managers, LZ adopted a dynamic systems approach [40] to understand the various interactions, roles, structures, and existing training and support available to practitioners and hostel staff in their support of older hostel residents with memory and cognitive problems. She systematically examined associations that emerged naturally between each important component, allowing pertinent overarching themes and subthemes to develop organically [36, 41]. The analysis revealed potential areas for enhanced collaboration between health and social care practitioners and hostel staff in navigating the healthcare system for hostel residents.

### Ethical considerations

London (Brighton and Sussex) National Research Ethics Service approved the study (reference: 21/LO/0541) on 6th September 2021. Participants gave oral and written informed consent and could withdraw from the study at any time. All interviews were transcribed anonymously to protect the confidentiality and integrity of participants.

### Reflexivity

LZ’s curiosity and personal interest in dementia and homelessness motivated her to explore the training and support needs of homelessness hostel staff who provide support to older hostel residents experiencing memory and cognitive problems. As Braun and Clarke’s [35] reflexive thematic analysis methodology emphasises, LZ studied these topics with self-awareness and transparency by recognising her preconceptions, prejudices, and personal experiences and kept a reflective journal on her thoughts. LZ sought support from co-authors to understand the English healthcare system better. Acknowledging her positionality as a white European and her lack of experience in working in the homelessness sector, she tried to remain open-minded, accepting participants’ different perspectives, and seeking validation from other researchers.

## Results

### Participant characteristics

From September to December 2021, 32 semi-structured interviews were conducted, 20 with women and 12 with men. Most participants were White British and had experience with memory loss and homelessness. Of the 32 participants, 17 were health and social care practitioners and 15 were staff and managers, with an average age of approximately 42 years old. The demographics of staff and health and social care practitioners are shown in Tables 1 and 2.

**Table 1:** Characteristics of HS and managers

Characteristic	Category	*n* (%) or mean (*SD*)
**Age**		42.2 (10.4)
**Gender**	Female	10 (66.7)
	Male	5 (33.3)
**Ethnicity**	White British	11 (73.3)
	Asian British	2 (13.3)
	White other	1 (6.7)
	Black British	1 (6.7)
**Job title**	Managers/deputy managers/team leaders	4 (26.6)
	Complex needs/health/specialist workers	4 (26.6)
	Support workers	4 (26.6)
	Project workers	3 (20.0)
**Time working in current role**	< 1 year	3 (20.0)
	1–3 years	7 (46.6)
	3–5 years	3 (20.0)
	5–10 years	1 (6.7)
	10 + years	1 (6.7)
**Time working in homelessness sector**	< 1 year	3 (20.0)
	1–3 years	2 (13.3)
	3–5 years	2 (13.3)
	5–10 years	5 (33.3)
	10 + years	2 (13.3)
	Did not say	1 (6.7)
**Past experience working with people with memory problems**	Yes	11 (73.3)
	No	3 (20.0)
	Did not specify	1 (6.7)

**Table 2:** Characteristics of health and social care practitioners

Characteristic	Category	*n* (%) or mean (*SD*)
**Age**		42.2 (10.4)
**Gender**	Female	10 (58.8)
	Male	7 (41.2)
**Ethnicity**	White British	15 (88.2)
	White other	1 (5.9)
	Asian other	1 (5.9)
**Professional role**	Specialist nurse	3 (17.6)
	Service manager	3 (17.6)
	Psychiatrist	3 (17.6)
	Psychologist	2 (11.8)
	Coach	1 (5.9)
	Geriatrician	1 (5.9)
	GP	1 (5.9)
	Housing commissioner	1 (5.9)
	Social worker	1 (5.9)
	Speech and language therapist	1 (5.9)
**Time working with memory problems**	1–3 years	1 (5.9)
	3–5 years	2 (11.8)
	5–10 years	3 (17.6)
	10 + years	11 (64.7)
**Time working in homelessness**	< 1 year	3 (17.6)
	1–3 years	1 (5.8)
	3–5 years	1 (5.8)
	5–10 years	3 (17.6)
	10 + years	9 (52.9)

### Qualitative findings

We identified three themes corresponding to the aims: a) *Emotional and psychoeducational support needs within* *hostel staff* *teams*, b) *Training and support needs to deliver a person-centred approach*, and c) *Support to facilitate collaboration with external agencies. *These themes and subthemes are shown in Table 3, with perspectives from the different stakeholder groups (i.e., health and social care practitioners as well as hostel staff and managers) discussed in the following paragraphs.

**Table 3:** Table of themes and differences between stakeholder groups

Themes	Subthemes	Difference in stakeholder contribution to subthemes	Respondents referenced per subtheme
*Emotional and psychoeducational support needs within* *hostel staff teams*	*Training on memory and cognitive problems and their associated responsive behaviours*	HSCP < HS	HS1, HS2, HS3, HS4, HS5, HS6, HS7, HS8, HS9, HS10, HS11, HS12, HS13, HS14, HS15 HSCP1, HSCP2, HSCP3, HSCP4, HSCP5, HSCP6, HSCP8, HSCP9, HSCP10, HSCP12, HSCP15, HSCP16, HSCP17
	*A reflective space for* *hostel staff to regulate their responses*	HSCP < HS	HS1, HS2, HS3, HS4, HS5, HS6, HS7, HS8, HS9, HS11, HS12, HS15 HSCP1, HSCP2, HSCP3, HSCP6, HSCP7, HSCP8, HSCP10, HSCP11, HSCP12, HSCP13,
*Training and support needs to deliver a person-centred approach*	*Support for* *hostel staff* *to better respond to distress behaviours*	HSCP = HS	HS1, HS2, HS3, HS4, HS5, HS6, HS7, HS8, HS9, HS10, HS15 HSCP1, HSCP2, HSCP3, HSCP4, HSCP5, HSCP6, HSCP7, HSCP10, HSCP12, HSCP13, HSCP14
	*Training to improve communication skills around memory and cognitive problems*	HSCP = HS	HS1, HS2, HS3, HS4, HS5, HS6, HS7, HS8, HS9, HS10, HS12, HS13, HS14 HSCP1, HSCP2, HSCP4, HSCP6, HSCP8, HSCP9, HSCP10, HSCP11, HSCP12, HSCP13, HSCP14, HSCP15, HSCP16
	S*upport to foster a protective space for hostel residents with memory and cognitive problems while respecting their independence*	HSCP < HS	HS1, HS2, HS3, HS4, HS5, HS6, HS7, HS8, HS9, HS10, HS11, HS12, HS13, HS14, HS15 HSCP1, HSCP2, HSCP4, HSCP5, HSCP6, HSCP7, HSCP8, HSCP9, HSCP10, HSCP11, HSCP12, HSCP13, HSCP15, HSCP16
*Support to facilitate collaboration with external agencies*	*Training and support to address barriers in accessing external resources and improve collaboration*	HSCP > HS	HS1, HS2, HS3, HS5, HS6, HS7, HS8, HS9, HS10, HS11, HS12, HS13, HS14, HS15 HSCP1, HSCP2, HSCP3, HSCP4, HSCP5, HSCP6, HSCP7, HSCP8, HSCP9, HSCP10, HSCP11, HSCP12, HSCP13, HSCP14, HSCP15, HSCP16, HSCP17
	*Support mechanisms to advocate for hostel residents with dual diagnosis and to assess their capacity*	HSCP > HS	HS2, HS3, HS4, HS6, HS8, HS9, HS12, HS15 HSCP1, HSCP2, HSCP3, HSCP4, HSCP5, HSCP6, HSCP7, HSCP8, HSCP9, HSCP10, HSCP11, HSCP12, HSCP13, HSCP14, HSCP15, HSCP16, HSCP17

*HSCP* Health and social care practitioners, *HS* Hostel staff, *HSCP* < *HS* indicates that hostel staff emphasized the subtheme more frequently than health and social care practitioners, *HSCP* > *HS* indicates that health and social care practitioners emphasized the subtheme more frequently than hostel staff, *HSCP* HS indicates that both groups emphasized the subtheme equally

### Theme 1: Emotional and psychoeducational support needs within hostel staff teams

This theme highlights the importance of emotional and practical support for hostel staff to meet the needs of older hostel residents with memory problems. Both health and social care practitioners and hostel staff underlined the need for hostel staff to get emotional support and learn about memory and cognitive problems to help hostel residents. Another hostel.

#### Subtheme 1: Training on memory and cognitive problems and their associated responsive behaviours

Hostel staff highlighted the need for professional knowledge and training to identify and recognise types and causes of memory and cognitive problems, to evaluate their severity, and to handle responsive behaviours. Hostel staff were more frequently involved in discussions about development needs for managing memory loss among hostel residents, reflecting their direct interactions with affected individuals. Hostel staff suggested educational group discussions on these topics, explaining that structured, regular discussions could help staff better understand and address hostel residents’ needs. As one staff member shared, “I think we should take time at least once a month to actually talk about clients in the groups. And let’s have a discussion on the kinds of memory problems. Let’s have a discussion on the client’s brain injuries” (HS1). Another emphasized the importance of communication skills and specific strategies for interacting with hostel residents, stating, “Some really basic knowledge of solution-based interventions. Ability to communicate and not make the situation worse. […], staying cognitively engaged. […] I think that is one of the things that alleviates agitation and anxiety, panic…” (HS2). One health and social care practitioner underlined the importance for hostel staff understanding both the medical history and personal life experiences, stating, “What’s the medical background? What life experiences have happened that may help us start to unpick some of this picture? And […] some clinical knowledge” (HSCP1).

#### Subtheme 2: A reflective space for hostel staff to regulate their responses

Hostel staff highlighted the challenging nature of the work due to the complex and overlooked needs of older hostel residents with memory and cognitive problems, necessitating reflective spaces to manage emotional responses. Hostel staff expressed worries about overwork, so they prioritised hostel residents who are easier to move out. However, one health and social care practitioner underscored the importance of values over targets stating, “They often feel like they’re in trouble or they’ve not met their targets […], usually they want to make a positive difference so kind of linking that, those values to whatever the intervention is will be quite important” (HSCP2). Hostel staff felt that they lacked sufficient opportunities to reflect on their emotions and proposed implementing protected time and reflective spaces where they could discuss their doubts and questions. After working with older persons with responsive behaviour, hostel staff recognised the need for reflective supervision. As one health and social care practitioner shared, “I think the staff themselves could maybe benefit with some supervision to kind of address some of the traumas […] of seeing people die in hostel beds and having to witness violence” (HSCP3). Another hostel staff emphasised the role of emotions in their work, explaining, “Being a bit more in tune with your emotions and being reflective I think is really key with working here” (HS3). Another hostel staff reflected on the emotional demands and the need to manage their own expectations while responding to residents with memory problems, stating, “It also is a constant battle over paperwork and trying to keep on top of things […]. You come to work and can’t complete your tasks because of how busy things can get. […]. Managing expectations comes with a change. But how do you address something that he won’t remember happened? So it’s just [support on] being ready to deal with those” (HS4).

### Theme 2: Training and support needs to deliver a person-centred approach

Difficulty engaging with cognitively impaired hostel residents and the need for specialised care to meet their complex needs while preserving safety and independence were challenging for hostel staff. Hostel staff described the need for complementary tools and frameworks to deliver a good person-centred approach.

#### Subtheme 1: Support for hostel staff to better respond to distress behaviours

To provide the appropriate support, staff wanted strategies that focus on the hostel residents’ well-being and preferences as well as ways to satisfy their unmet needs. Health and social care practitioners recommended helpful tools and support package to enhance hostel residents’ independence and reduce vulnerability. As one health and social care practitioner explained, “I think care packages, you know practical support through adult social care can make a huge difference to someone’s independence. Even if it is extra help, you know, it frees them up to defend themselves or to not be embarrassed or picked on” (HSCP4). Another health and social practitioner emphasized the value of understanding residents’ life stories to address behaviours, explaining, “Having a life story, perhaps […] around the person’s behaviours, because that’s why they’re behaving that way, because the person might have been on night shift or something. […] If the person’s having challenging behaviours, how to sort of handle that” (HSCP5). Hostel staff also highlighted the importance of training to handle physically challenging situations. As one staff member shared, “something that should be included is […] breakaway training […]. Being able to get out of a physically challenging situation” (HS5). Hostel staff acknowledged the necessity for multi-disciplinary training meetings to better evaluate hostel residents’ capabilities and needs, as illustrated by one staff member, “We have to have, like, […] multi-disciplinary meeting […] to assess whether, you know, the client can get his own food, wash his own laundry and that kind of stuff” (HS6).

#### Subtheme 2: Training to improve communication skills around memory and cognitive problems

Health and social care practitioners emphasised that poor communication skills between hostel staff and hostel residents often prevents hostel residents’ needs from being met, as hostel residents require clear and consistent interactions to understand and respond to their environment. A health and social care practitioner suggested some prompts to facilitate communication with hostel residents: “Orientate the person with a clock, a diary, a watch, the way you do your keywork pictures [which are visual prompts used in case management to aid memory and orientation], […] writing things up for them and breaking things down for people with brain injuries. […] And talking mats and flow charts” (HSCP6). Another hostel staff member mentioned the need for training to communicate with the type of language that the hostel residents understand explaining, “When you’re working with somebody who has memory problems, then it’s even more important to be focused because they’ll pick up on other cues, body language and stuff like that.” (HS7). Another hostel staff emphasised the need for communication strategies with residents, stating, “How to work with them on a daily basis, like […] your attitude to them, […] the way you can speak to them, to make them feel a bit more comfortable. […] some tips about communication with these clients” (HS8).

#### Subtheme 3: Support to foster a protective space for hostel residents with memory and cognitive problems while respecting their independence

Hostel staff frequently complained about a lack of training and assistance on how to adapt their responses and modify the environment to create a secure, comfortable, hostel resident-centred environment, and to promote a more therapeutic environment for hostel residents. They discovered that providing opportunities for people with lived experience of memory problems to express themselves helped overcome this obstacle: “Maybe incorporating people who are actually suffering from memory issues to give their [perspective and] to say, look, this is this is what I’m experiencing.” (HS7). Furthermore, hostel staff emphasised the need for training to protect memory-impaired hostel residents from exploitation by other fellow hostel residents while encouraging socialisation and independence. An ideal scenario was described by a hostel staff member, “Older, more vulnerable clients […] would have swipe card to be able to access […] communal space there so […] staff could cook something with them” (HS9). They also discussed ways individuals with memory and cognitive problems could explore the hostel or find their way, “Like colours of the corridors and how much using signage to try and like direct people […] the right way.” (HS10) and “Putting names and address cards in their jackets […], putting signs they’re familiar with […] to jog their memory” (HS5). A health and social care practitioner highlighted the challenges hostel staff face in transitioning residents with memory and cognitive problems to suitable placements while ensuring their safety and independence, stating, “There was a feeling by the staff in the hostels that they were probably after his money and […] it kind of led to safeguarding issue. It’s been a big struggle to find somewhere for him to go after hostel” (HSCP7). Another health and social care practitioner further elaborated on the complexities of providing adequate support, “He was getting more and more exploited in the hostel. […] We had a care package [but] he didn’t really want to accept any care. So, we were really stuck in this battle with him” (HSCP8).

### Theme 3: Support to facilitate collaboration with external agencies

Hostel staff questioned the accessibility of external agencies, such as healthcare providers and social services, as well as the necessity of training to navigate these interactions effectively. This training would cover both practical communication skills for connecting with these agencies and increasing awareness of the services they offer, supporting hostel staff in advocating more effectively for hostel residents’ comprehensive needs. This was particularly in the context of feeling that often they were trying to gain additional support or specialist assessment, for example from specialist alcohol services or memory assessment teams. Collaboration between hostel staff and external agencies may benefit hostel residents with memory and cognitive problems by allowing hostel staff to access external resources and share insights on hostel residents’ everyday behaviours. While hostel staff often advocate for and support residents in these interactions, the services are ultimately intended for the residents, who may not be able to access them successfully without this support. They stressed the need for a holistic viewpoint to assess competence and access support mechanisms to advocate for hostel residents’ independence through the right communication pathways, including collaborative decision-making and information sharing about hostel residents between different agencies.

#### Subtheme 1: Training and support to address barriers in accessing external resources and improve collaboration

Hostel staff underlined the necessity of training to utilise up-to-date professional language to obtain health and social care practitioner information to deliver good service. They argued that collaboration between hostel staff and health and social care practitioners may enhance understanding about different available pathways and help to create a more holistic and complete hostel resident diagnosis. For instance, one health and social care practitioner expressed the need for exchange of information about hostel residents between different agencies involved in providing services to people experiencing homelessness, stating, “Additional information from […] medical or health professionals to help fill in the blanks that that person might not be able to tell” (HSCP9). Health and social care practitioners also recommended accessing dementia training directly from them and invited hostel staff to bring case studies to discuss as one stated, “Simulations and role play and so we’ve done dementia training […] having a kind of regular thing, where people can come back and bring back cases and discuss what people might have done well and what people might do different” (HSCP10). Hostel staff highlighted the difficulties they face in being taken seriously by external professionals, which can hinder collaboration. One hostel staff member explained, “It just feels whenever I go to the doctors with him, […] I am just ignored whatever we want to say […] but it just seems to be [that] the doctors don’t take us seriously” (HS11). Hostel staff also expressed that training to improve communication skills is necessary for bringing other services inside the hostel as one hostel staff noted, “So more of a multidisciplinary team. […] trying to incorporate more into our team is bringing everything in-house” (HS6). This would help foster a more inclusive and collaborative support system for hostel residents with complex needs.

#### Subtheme 2: Support mechanisms to advocate for hostel residents with dual diagnosis and to assess their capacity

Hostel staff reported that hostel residents are frequently not viewed beyond their alcohol and substance use problems. They also emphasised the need for support mechanisms, specifically strategies and resources, with collaborating external agencies to eliminate dual diagnosis stigma. Health and social care practitioners emphasised hostel staff needed training to assess hostel residents’ capacity, which means determining their sobriety status and distinguishing between cognitive problems and substance use. A health and social care practitioner noted, “When there’s drugs or alcohol involved […] they don’t take into consideration that might be a symptom of memory loss or cognitive issues or choose to see them as a dual diagnosis” (HSCP4). Furthermore, a health and social care practitioner recommended training on acknowledging hostel residents’ strengths beyond drinking by stating, “One of the things that’s really helpful with the trauma informed approach is thinking about strengths, […] to view that person through a competence lens […] humanising people that we see beyond the drinking” (HSCP2). Capacity assessments were noted as a particular challenge as a health and social care practitioner noted that external services tend to misdiagnose or neglect to diagnose hostel residents who drink and that capacity assessments are not addressed, saying, “To get anyone properly assessed in regards to their cognition is really difficult in hospital […] I’ve had to ask XXX to do cognitive assessments, because they just refuse to” (HSCP11). In addition, health and social care practitioners suggested using another form of communication to evaluate capacity, as one mentioned, “The way we delivered that information to him needed to be different. So, it wasn’t just verbal. It was written […] not just closed questions, but also writing things down” (HSCP12). Hostel staff also highlighted the need for support mechanisms to enhance their skills for capacity assessment and to be taken more seriously by doctors and external professionals. One hostel staff noted, “Some specific support mechanisms, […] regular updates of resources and check ins and debriefs and maybe to get some more reflective practice with a with a supervisor that will say, […] we all need to be on the same page and everybody work on this direction” (HS2). Both hostel staff and health and social care practitioners emphasised the importance of collaborative efforts, comprehensive training, and advocacy tools to address the complex needs of hostel residents with dual diagnoses and enhance overall support.

## Discussion

### Main findings

The study aimed to investigate the training and support that hostel staff require to meet the needs of older hostel residents experiencing memory and cognitive problems, and thus enhance hostel resident quality of life and well-being, considering what additional knowledge, skills, and support hostel staff need to achieve this. We identified the need for a multifaceted approach to hostel staff support, with potential to ameliorate their ability to assist hostel residents, provide a better quality of life, and facilitate appropriate transitions from hostels.

Our study highlights the critical need for hostel staff to receive structured, reflective, emotional and psychoeducational support to navigate the challenges of their work and provide high-quality support, which aligns with existing research on reflective practice groups [42]. This directly addresses our aims by pinpointing the types of support that can mitigate feelings of being overwhelmed and boost confidence in working with hostel residents. The Positive Approach to Care framework supports this by providing techniques that improve hostel staff communication with memory-impaired hostel residents, fostering trust and empowering hostel residents to communicate their needs in a way that reduces the emotional strain on hostel staff while supporting high quality care [26, 27]. Existing research has highlighted that training for staff to find meaning in their roles and using Psychologically Informed Environments techniques specifically supports the need for reflective spaces where hostel staff can manage emotional response effectively [43], reducing feelings of being overwhelmed [21], and boosting confidence in engaging with hostel residents, particularly those with complex trauma [44].

Our findings also highlight the need for an approach which incorporates understanding of the needs of older hostel residents with memory and cognitive problems and the associated responsive behaviours [17]. This aligns with the Person-Centred Care approach, which emphasizes adapting communication and care practices to the unique needs of each individual, promoting a supportive environment that values hostel residents’ backgrounds and preferences [29, 30]. The Positive Approach to Care framework further reinforces this need by promoting communication strategies that enhance hostel residents’ autonomy and sense of control, fostering greater independence [27]. Dementia specific training can further change attitudes and raise awareness of the needs of older people experiencing memory problems [45], a finding that aligns with our study’s emphasis on enhancing hostel staff skills in communication and support. Our findings align with existing literature, which suggests that paying attention to humour and gestures [46] and using strategies like charts and clocks as suggested by health and social care practitioners, can increase independence and communication among people with memory and cognitive problems [47], underscoring the relevance of integrated training for hostel staff in our study’s context. Stevens-Roseman & Leung [30] discussed the importance of using active listening, ways to interact with memory-impaired and emotionally and physically distressed hostel residents, and ways to help hostel residents express their thoughts and feelings in care settings for older adults. Ripich et al. [48] suggested using verbal and nonverbal methods to improve communication. These approaches [30, 48] have been used in dementia care and homelessness support but to the authors’ knowledge have never been investigated together. This directly ties to our study’s aim by highlighting the need for training strategies that incorporate elements from both dementia care and homelessness support. Furthermore, our study underscores that there is a specific need for hostel staff training tailored to working with people experiencing memory and cognitive problems in homelessness settings, focusing on enhancing communication and improving hostel staff-resident interactions as key aspects of high-quality support.

Difficult or limited communication between hostel staff and external organisations often leads to a loss of holistic information about hostel residents experiencing memory and cognitive problems, who are often misdiagnosed with substance use problems. The Psychologically Informed Environment approach could enhance service delivery by fostering a broader awareness of memory and cognitive issues beyond substance use, supporting hostel staff in their advocacy for appropriate hostel resident care [44]. Our findings highlight the lack of support mechanisms for hostel staff to advocate for hostel residents’ cognition and memory problems [16]. Our study found that collaboration with external services and having a good understanding of the specific nature of hostel residents’ memory and cognitive problems could create mutual understanding and foster appropriate joint care. Aligned with earlier research [49], hostel staff in our study emphasised the need for better collaboration with external services to better support hostel residents. Our study highlights the importance of a holistic approach and the role of guidance from health services in addressing stigma and misinformation, enabling them to offer comprehensive and collaborative treatment which supports earlier research [24]. Furthermore, Interprofessional Education can promote holistic learning and collaborative care, helping reduce stigma and ensuring hostel residents’ cognitive needs are understood and respected [24]. This finding underscores the importance of external collaboration in enhancing the ability of hostel staff to meet hostel residents’ needs.

### Strengths and limitations

Focusing on homelessness hostels in England limits the generalisability of findings due to variations in healthcare systems and support networks. This geographic restriction hinders the transferability of the findings to hostels in other areas [50]. Additionally, (anonymised) did not conduct the interviews herself, potentially introducing bias. Consequently, the analysis may lack the depth of direct dialogue, including nonverbal cues (e.g., tone of voice), and an opportunity to seek clarification. However, one could argue that having the interviews conducted by coauthors also increases independence in analysis, as (anonymised) was not influenced by direct interactions, emotions, or nonverbal cues present during interviews. This separation in tasks between data collection and analysis allowed (anonymised) to approach the data with fresh eyes, potentially reducing personal biases and leading to a more objective interpretation. Since hostel staff training and support were one aspect of the interviews but not the main focus of the primary analysis, the interview questions may not have been tailored only to the aims of the present study, and this may have affected the interpretation and selection of relevant information. Although all 32 participants contributed to the dataset, space constraints required selecting only the most illustrative quotations. To enhance transparency, a column in Table 3 identifying contributors to each subtheme was included, ensuring the absence of direct quotes does not imply a lack of contribution to the thematic analysis. The present study relied on participants’ self-reported experiences and future research may benefit from additional observational methods to enrich the understanding of hostel staff-resident interactions and engagement with external services.

### Implications

This study emphasises the critical role of comprehensive training and emotional support for hostel staff working with older hostel residents who experience cognitive and memory challenges. Our findings suggest that it may be useful to develop interventions informed by the Psychologically Informed Environments, Person-Centred Care, Positive Approach to Care, Interprofessional Education frameworks to provide hostel staff the tools to manage responsive behaviours, communicate effectively, and offer holistic support. While each framework is adapted for dementia or homelessness, no existing approach has successfully integrated all these frameworks together. Therefore, to address both cognitive decline and homelessness, it might be necessary to find a way to combine all these approaches in a complementary way. Importantly, the findings from this study have been used to inform the co-production of the HOME intervention – a training and support intervention for hostel staff, now being tested in a feasibility study. The intervention includes education on the multiple causes and impacts of memory problems, understanding mental capacity, communication strategies, person-centred approaches, managing distressed behaviours, promoting social interaction, and communicating with external agencies regarding memory and other cognitive problems. Training and support for hostel staff would benefit from including structured emotional support meetings and reflective practice groups, as their work is demanding and emotionally challenging. Such sessions could provide hostel staff with tools for emotional coping, which may improve their resilience [42]. Training focused on enhancing hostel staff-resident communication using person-centred techniques, such as building trust, empathy, and understanding hostel residents’ backgrounds is important to improving care [31, 51]. Specific communication strategies may also be beneficial for hostel staff training and support. The Psychologically Informed Environments approach can enhance service delivery by raising awareness of memory and cognitive problems beyond substance use and fostering a holistic assessment [44]. Integrating these approaches requires creative collaborative learning platforms, which would bring together the traditionally separate fields of dementia care and homelessness support with partnerships across disciplines (e.g., gerontology, dementia, health and social care, homelessness). Involving hostel residents themselves, especially those with lived experiences of homelessness and memory and cognitive problems, alongside health and social care practitioners, could be essential in creating a new standardised assessment framework tailored to this population’s specific needs. This shift to a person-centred approach empowers hostel residents to actively participate in their support, potentially reducing responsive behaviours [46]. However, such involvement would also provide hostel staff with insights that enhance their ability to address hostel residents’ needs effectively. This is consistent with the broader aim of improving hostel staff skills and knowledge as outlined in NICE NG214 [52]. Lastly, building on the NICE guideline NG214 which emphasises hostel staff well-being and multidisciplinary working [52], resource allocation, such as time management and hostel organisation to bring mental health professionals into hostel staff meetings could be necessary to support hostel staff emotionally.

## Conclusions

This study offers insight into the crucial support and training needs for hostel staff in homelessness hostels who support people experiencing memory and cognitive problems. Our findings highlight the importance of equipping hostel staff with the knowledge, skills, and emotional support required to address these challenges effectively. Our study provides a unique and inclusive perspective by addressing two previously separate areas, homelessness hostels and memory and cognitive problems. This study recommends future research on developing and evaluating tailored training interventions for hostel staff, focusing on practical skills and strategies to enhance their capacity to support hostel residents effectively.

## Supplementary Information

Additional file 1. Interview topic guide for HSCP and HS and managers.

## Data Availability

The qualitative data used and analysed during the current study are available from the corresponding author on reasonable request.

## Abbreviations

ADRD: Alzheimer's Disease and Related Dementias
ETHOS: European Typology of Homelessness and Housing Exclusion
HS: Hostel staff
HSCP: Health and Social Care Practitioners
NHS: National Health Service
NIHR: National Institute for Health and Care Research
NICE: National Institute for Health and Care Excellence
UK: United Kingdom
