# Supplementary material for: What are the training and support needs for homelessness hostel staff supporting older residents with memory and cognitive problems? A qualitative study
Source: BMC Health Serv Res. 2025 Jan 21;25:114. doi: 10.1186/s12913-025-12246-2 (PMC11752725; doi:10.1186/s12913-025-12246-2)
Supplement: Supplementary file 1 — Additional file 1. Interview topic guide for HSCP and HS and managers. [file 12913_2025_12246_MOESM1_ESM.docx]

**Supplementary material**

**Additional file 1** Interview topic guide for HSCP and HS and managers

| 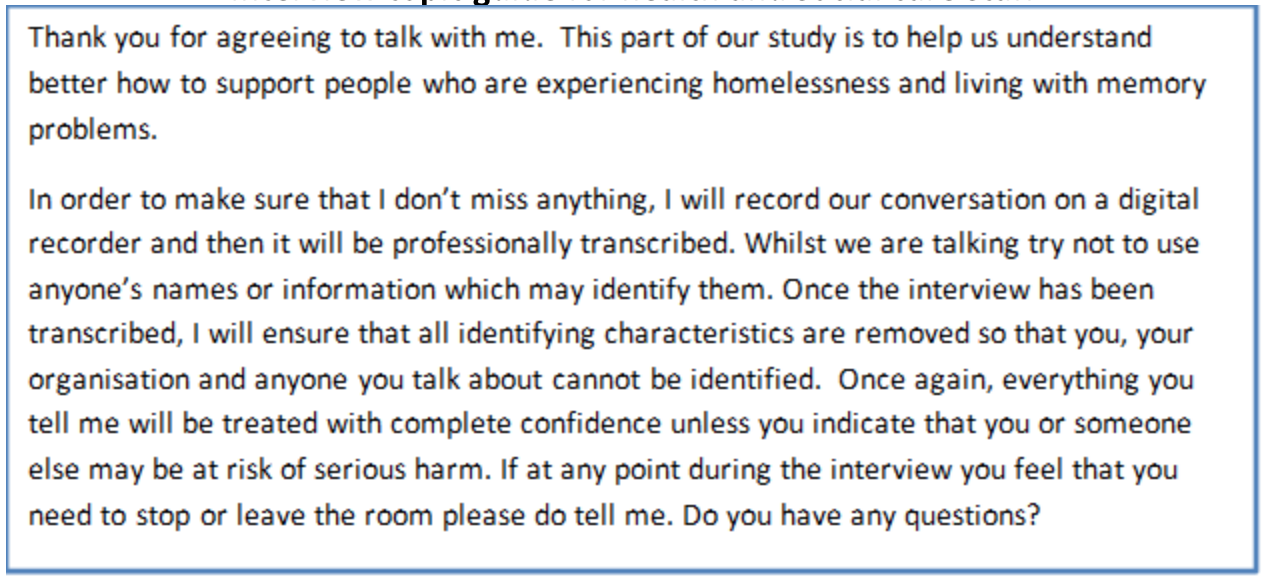  **To start, could you tell me a little about your work? How are you involved with caring for people experiencing homelessness and or memory problems?**   - Can you describe (without using name) a particular older person who you have worked with and what their difficulties were? - How did you make sense of and understand their difficulties? - How did their memory difficulties impact upon their day to day life and wellbeing? - How did their memory difficulties get in their way of moving to or being in settled accommodation?   **What do you see as the main challenges in supporting older people with memory problems who are experiencing homelessness and what might help**   - Challenges at a service level? - Challenges at a team level? - Challenges at an individual staff level? - Challenges related to the individual with memory problems?   **What do you think ‘best practice’ would look like in supporting older people with memory problems who are experiencing homelessness?**   - What would good health care look like? - What would good social care look like? - What would help people to move on from homelessness / hostel / temporary accommodation?   **What do you think would be important positive outcomes for older people with memory problems who are experiencing homelessness?**   - What might this look like in practice? - What / who might help them to achieve these outcomes? - What factors might get in the way of these positive outcomes   **We want to develop a support intervention for hostel staff supporting older residents with memory problems. What do you think it would be important to include?**   - What areas or topics would it be important to address? - Is there any specific training that would be useful? - What may help/make it harder for frontline staff to put support strategies and new learning into practice?   **What knowledge and skills might frontline staff in homelessness organisations need to support older people experiencing memory problems?**   - What could help them to build these skills? - What could we learn from approaches in other related areas (e.g. dementia care, support in homelessness, drug and alcohol support etc)     **Thank you - Is there anything else you would like to add?** |
| --- |
